# Supplementary material for: A flexible cross-platform single-cell data processing pipeline
Source: Nat Commun. 2022 Nov 11;13:6847. doi: 10.1038/s41467-022-34681-z (PMC9652453; doi:10.1038/s41467-022-34681-z)
Supplement: Supplementary file 3 — Description of Additional Supplementary Files [file 41467_2022_34681_MOESM3_ESM.pdf]

Supplementary Data

Zip files for samples of output are provided for each technology as an example of the processed data returned by UniverSC. This data can also be used to reproduce the results in Figure 2 and Figure 3.

Data S1: Results for UniverSC 1.2.0 on Chromium version 3 data (Zheng et al., 2017).
Data S2: Results for Cell Ranger 3.0.2 on Chromium version 3 data (Zheng et al., 2017).
Data S3: Results for UniverSC 1.2.0 on Drop-seq data (Macosko et al., 2015).
Data S4: Results for dropSeqPipe 0.6 on Drop-seq data (Macosko et al., 2015).
Data S5: Results for UniverSC 1.2.0 on ICELL8 data (Goldstein et al., 2017).
Data S6: Results for CogentAP 1.0 on ICELL8 data (Goldsein et al., 2017).
Data S7: Results for UniverSC 1.2.0 on SmartSeq3 data (Hagemann-Jensen, et al., 2020).
Data S8: Results for zUMIs 2.9.7 on SmartSeq3 data (Hagemann-Jensen, et al., 2020).
Data S9: GBM of Chromium version 2 data (Mereu et al., 2020) processed via UniverSC.
Data S10: GBM of SmartSeq2 data (Mereu et al., 2020) processed via UniverSC 1.2.0.
Data S11: GBM of SmartSeq2 data (Mereu et al., 2020) processed via zUMIs 2.9.7.
