# Supplementary material for: A flexible cross-platform single-cell data processing pipeline
Source: Nat Commun. 2022 Nov 11;13:6847. doi: 10.1038/s41467-022-34681-z (PMC9652453; doi:10.1038/s41467-022-34681-z)
Supplement: Supplementary file 2 — Editorial Assessment Report [file 41467_2022_34681_MOESM2_ESM.pdf]

nature portfolio

GUIDED OPEN ACCESS

nature methods nature communications communications biology

Editorial Assessment Report

Dear Dr Minoda,

Thank you again for choosing to submit your manuscript using the Guided Open Access pilot at the Nature Portfolio. As part of this process, our editorial team has considered your paper for three of our journals with strong interest in publishing in your field: *Nature Methods*, *Nature Communications*, and *Communications Biology*.

Your manuscript entitled "UniverSC: a flexible cross-platform single-cell data processing pipeline" has now been reviewed by 3 experts in web-based platforms and computation tools for single cell analysis, whose comments are included in the attached Editorial Assessment Report. As part of the Guided Open Access pilot, editors from all 3 journals have discussed the reviewer reports and the manuscript's suitability for our journals. After careful evaluation, our editorial recommendation is to revise the manuscript and submit back through the Guided Open Access submission portal for consideration at *Communications Biology* or *Nature Communications*. Provided the revisions satisfy all technical and editorial concerns, *Communications Biology* is very interested in publishing your manuscript. Please see details in the attached Editorial Assessment Report.

In brief, for publication in Communications Biology, we would require you to address the referee concerns regarding adapting the method to work with more pipelines and making it compatible with combinatorial indexing technologies. For consideration in Nature Communications, the editors would request all of these changes, as well as additional implementation of a GUI that interfaces with a light-weight version that can be installed locally, or with secure cloud-based server, in order to expand its utility.

Please note that the Editorial Assessment Report is a standalone document that contains an editorial evaluation, recommendation and portable peer advice to help you navigate and interpret the reviewers' reports. It also provides guidance for adhering to best practice with regard to transparency and reproducibility, for example on the issue of sharing data. We have also included information about data accessibility and reproducibility, which we hope you find useful.

Should you have any questions about the recommended journals or would like advice on the revisions, you can contact me directly and I will be happy to assist. We look forward to receiving the revised version of your manuscript.

Yours sincerely,

Anam Akhtar

--

Dr Anam Akhtar

Associate Editor, *Communications Biology*

On behalf of the Guided OA editorial team

nature portfolio
GUIDED OPEN ACCESS
nature methods nature communications communications biology

Contents of this Report

- **Manuscript overview**: details about your manuscript and the editorial team.
- **Manuscript assessment**: personalised recommendation from the editors.
- **About the editorial process**: an overview of the Guided Open Access process.
- **Annotated reviewer comments**: the referee reports with comments from the editors.
- **Open research evaluation**: advice for adhering to best reproducibility practices.

Manuscript overview			
**Manuscript number** GUIDEDOA-21-000444	**Submission date** 15 February 2021		**Decision date** 9 April 2021
**Title**	UniverSC: a flexible cross-platform single-cell data processing pipeline	**Corresponding author**	Aki Minoda<br>ORCID: 0000-0002-2927-5791
**Preprint information**	Preprint deposited at bioRxiv https://www.biorxiv.org/content/10.1101/2021.01.19.427209v1	**Peer review type**	Single-blind
**Editorial Assessment Team**	**Primary editor: Anam Akhtar**<br>**Home Journal:** *Communications Biology*, ORCID: 0000-0002-8820-8468<br>**Editorial team members:**<br>Lin Tang, *Nature Methods*<br>Doaa Megahed, *Nature Communications*, ORCID: 0000-0002-3455-2992		
**About your primary editor**	Anam received her PhD in Biomedical Sciences from Middlesex University, designing targeted liposomal constructs for selective drug delivery to HPV infected cancers. She also has research experience in genetics and the design of polymer and inorganic nanoparticles and their applications. Anam joined the editorial team of Communications Biology in July 2019 and is based in London office.		

Manuscript assessment and recommendation	
**Editor's summary of the manuscript and overall assessment**	Aki Minoda and colleagues develop UniverSC, a shell utility that works as a wrapper for Cell Ranger (10x Genomics), which can handle datasets generated by a range of different UMI-based single-cell technologies. It carries out its entire process in seven steps: 1. basic input curation, 2. pipeline-specific modifications, 3. modify R1 file to have 16 bp barcode and 10 bp or 12 bp UMI, 4. get barcode/UMI length and whitelist file based on the given technology, 5. modify selected whitelist barcodes to be 16 bp, 6. (if needed) overwrite the cellranger whitelist file in place, 7. run Cell Ranger. Its current version has pre-set parameters for 19 technologies. A few testing datasets are provided. They use it to analyze published test datasets from human cell lines (10x Genomics (version 3), DropSeq, and ICELL) which leads to highly similar results when compared to existing platform-specific pipelines.<br><br>The editors felt that the work was well-developed and the task at hand, i.e. generating a tool that enables analysis across single cell technologies, is very important. The methodology, however, was not sufficiently new, nor the performance advantage sufficient to meet the criteria for *Nature Methods*. The referees also had some serious concerns over its potential benefit, difficulty in installation and running and other existing similar tools.<br><br>In summary, for a resubmission to *Communications Biology* or *Nature Communication*s, the minimum requirement would be to adapt UniverSC to work with more pipelines (mainly those requiring less memory) and also make it compatible with combinatorial indexing technologies as requested by Reviewer 1.

Editorial assessment overview

Journal	Decision	Assessment
Nature Methods	Not feasible to meet the editorial requirements	Neither the conceptual advance and advance in performance demonstrated are sufficient for publication in *Nature Methods.*
Nature Communications	Major revisions needed	All revisions requested by *Communications Biology*, plus the implementation of a GUI that interfaces with a light-weight version that can be installed locally, or with secure cloud-based server.
Communications Biology	Major revisions needed	Adapting the method to work with more pipelines and making it compatible with combinatorial indexing technologies is needed for publication.

**Editor's recommendation**	***Option 1*: Revise for consideration at Communications Biology**<br>We ask that you expand the utility of UniverSC to combinatorial indexing technologies, adapt it to work with more pipelines and also distinguish from STARsolo. While these revisions are required, we do not require the development of a GUI for UniverSC. See the annotated comments below.<br><br>***Option 2*: Revise and submit to Nature Communications**<br>In addition to addressing the comments requested for publication at Communications Biology (see above), publication at Nature Communications requires that the authors make UniverSC accessible to a wider range of users, particularly those unfamiliar with command lines for the Unix systems. That requires implementing a GUI to facilitate user interaction. In addition, UniverSC can either be implemented as a much lighter version to allow its use on an average Linux, Mac or PC laptop, or it can be run on the cloud while allowing users the option to easily and securely upload their data for analysis and download the results. The level of data transfer/storage security should be compliant with widely accepted standards for non-human data at least.<br><br>**Please state in your cover letter which journal you have revised for.**
**Next steps**	If you would like to follow our recommendation, when you are ready you can upload the revised manuscript, along with your point-by-point response to the reviewer's reports and editorial advice **here*.** If you need assistance with our manuscript tracking system, please contact Adam Lipkin, our Nature Portfolio Guided OA support specialist at guidedOA@nature.com.<br>**This URL links to your confidential home page and associated information about manuscripts you may have submitted, or that you are reviewing for us.*

About the editorial process

By selecting the Nature Portfolio Guided Open Access option, your manuscript was assessed for suitability in three of our titles that provide venues for publication of high-quality work across the spectrum of methods development research: *Nature Methods, Nature Communications*, and *Communications Biology*. For more information about Guided Open Access, please see here.

Collaborative editorial assessment

Your editorial team discussed the manuscript to determine its suitability for the Nature Portfolio Guided OA pilot. Our assessment of your manuscript takes into account several factors, including whether the work meets the **technical standard** of the Nature Portfolio and whether the findings are of **immediate significance** to the readership of at least one of the participating journals in the Nature Portfolio Guided Open Access methods cluster.

Peer review

Experts were asked to evaluate the following aspects of your manuscript:

Novelty in comparison to prior publications;

Likely audience of researchers in terms of broad fields of study and size;

Potential impact of the study on the immediate or wider research field;

Evidence for the claims and whether additional experiments or analyses could feasibly strengthen the evidence;

Methodological detail and whether the manuscript is reproducible as written;

Appropriateness of the literature review.

Editorial evaluation of reviews

Your editorial team discussed the potential suitability of your manuscript for each of the participating journals. They then discussed the revisions necessary in order for the work to be published, keeping each journal's specific editorial criteria in mind.

Journals in the Nature portfolio will support authors wishing to transfer their reviews and (where reviewers agree) the reviewers' identities to journals outside of Springer Nature. For any questions about review portability, please contact our editorial office: guidedoa@nature.com

nature portfolio

GUIDED OPEN ACCESS

nature methods · nature communications · communications biology

Annotated Reviewer Reports

The editor has included some additional comments on the specific points raised by the reviewers below. However, please note that all points should be addressed in a revision, even if the editor has not specifically commented on them.

Reviewer #1

Report received: 12 March 2021

**Reviewer #1**	This reviewer has not chosen to waive anonymity. The reviewer's identity can only be shared with representatives of an established journal editorial office.
**Reviewer #1 expertise** *Summarised by the editor*	This reviewer is an expert in tools for analysis of single molecule sequencing data
**Editor's comments about this review**	All points raised by this reviewer must be addressed. The requirement of GUI is not mandatory for consideration at Communications Biology. The reviewer raises valid suggestions on making UniverSC more user-friendly and of useful to the community.

Reviewer #1 comments

**Overview**	The authors have developed UniverSC, a wrapper for the 10X Genomics CellRanger software that is compatible with 19 different single cell technologies. UniverSC modifies the cell barcode and Unique Molecular Index (UMI) to be compatible with CellRanger, thus enabling users to generate gene expression matrices from a variety of single cell technologies. The authors demonstrate UniverSC on datasets generated using 10X genomics, Drop-seq, and ICELL8 technologies and benchmark UniverSC results against existing pipelines for those technologies.<br><br>As single cell sequencing becomes cheaper and more popular, democratizing and simplifying the analysis is critical. I applaud the authors for attempting to generate a tool that can easily enable analysis across single cell technologies. However, while the concept of UniverSC certainly has broad potential, I'm not sure that the UniverSC tool as currently implemented makes single cell analysis significantly easier over existing tools such as DropEst or Kallisto/Bustools, which are other single cell analysis pipelines that can be configured to work with multiple single cell technologies.

Specific comments

#	Reviewer comment	Editorial comment

**1**	**Comment on Impact:**<br>As it currently stands, I don't think UniverSC has a significant impact over existing tools for processing single cell RNA-seq data from multiple technologies such as Kallisto/Bustools and DropEst. While UniverSC has pre-configured settings for each technology, installing and running UniverSC still requires some amount of familiarity with Unix systems. I'm not sure it really is that much more effort to set the configurations for DropEst or Kallisto/Bustools vs using UniverSC. Additionally, while it was great to see a Docker container provided for users who want to run analyses on their laptops or home computers, the fact that UniverSC uses CellRanger means that most users can't realistically use UniverSC on their laptops or home machines since CellRanger has a pretty large memory requirement (32 gb RAM) and is quite slow.	
**2**	**Critical improvements**<br><br>1. I think a critical improvement to UniverSC could be adapting it to work with not only CellRanger, but also DropEst and Kallisto/Bustools, each of which improves on CellRanger in a number of ways. Giving users the option to use any of these three analysis pipelines across a variety of single cell technologies would enable UniverSC to be a powerful, universal platform for analysis. I think adapting UniverSC to work with Kallisto/Bustools, or another lightweight pipeline like Salmon-Alevin-fry, is especially critical as these methods have much smaller memory footprints and are much faster than CellRanger, enabling users to realistically process single cell data on their laptops or home machines.<br><br>2. Another critical improvement is making sure UniverSC is compatible with combinatorial indexing technologies such as Split-Seq and sci-RNA-seq. While the authors state that they are currently working on incorporating these technologies, ensuring that UniverSC works with these technologies before publication is important. A commercially produced Split-Seq kit has recently been released and it seems likely that a large fraction of future single cell datasets will be generated with these combinatorial indexing technologies.	**NatComms: Given that accessibility to a wide range of users, most importantly, novices in the analysis of single cell data and non-Unix users, UniverSC should be easy and light-enough to install locally on a standard MAC or PC. Alternatively, users should be provided clear and easy instructions on how the docker container can be setup on a variety of cloud computing services (see more below).**<br><br>**Please adapt UniverSC to work with other pipelines, particularly the lightweight pipeline as suggested to enable processing the data on laptops or home computers.**<br><br>**We also require that you please make UniverSC compatible with combinatorial indexing technologies mentioned here.**
**3**	**Optional suggestions**	**NatComms: Whether implemented to run locally on a**

	1. Making a graphical user interface for UniverSC would do quite a bit to enable scientists who are not familiar with Unix systems to process single cell datasets. However, I understand that making GUIs can be extremely time consuming.<br><br>2. Demonstrating the unique utility of UniverSC in a main figure would be really helpful. For example, if the authors could show that by re-processing all of these datasets with UniverSC, they were able to reduce the strength of technology specific batch effects, that would be a more convincing demonstration of UniverSC's impact.	**Linux, MAC or PC or on the cloud, a user interface should be provided. This is not required for Communications Biology.**<br><br>**We do ask for this figure to clearly demonstrate UniverSC's impact.**
**4**	**Comments on Reproducibility**:<br><br>The authors provide an easy to use shell script and Docker container for reproducibility, which is great to see.<br><br>Some minor comments on the figure layout and reporting summary:<br><br>1. The way Figure 2 is currently laid out is a bit confusing. I'm currently not sure how to interpret the plots in each column as the descriptions in the figure legends are a bit sparse. I think it would be helpful to arrange the panels so that each column is a panel, enabling a more thorough description of each type of plot in the figure legends.<br><br>2. I'm also not sure I understand the correlation plot in the first column of Figure 2. If this is a direct comparison of gene expression matrices, why are there so few data points?<br><br>3. The github link in the reporting summary is broken.	**Please address all these comments and ensure that the github link in Reporting Summary is working.**

**Reviewer #2**<br>*Report received:* 12 March 2021	
**Reviewer #2**	This reviewer has not chosen to waive anonymity. The reviewer's identity can only be shared with representatives of an established journal editorial office.
**Reviewer #2 expertise**<br>*Summarised by the editor*	This reviewer is an expert on web-based platforms for single cell analysis

**Editor's comments about this review**	This reviewer questions the novelty of the UniverSC. While the reviewer has given a very short report, they raise an important point on how another program, STARsolo could be a competitor, performing similar task. We would like you to clearly distinguish UniverSC from STARsolo and demonstrate UniverSC's superiority/ advantages over it.
**Reviewer #2 comments**	
**Overview**	Kelly et al., present a pipeline called UniverSC that aims to wrap preprocessing pipelines from several single-cell protocols in a single scripted tool. UniverSC is mainly wrapping configurations files around the CellRanger pipeline, so that multiple protocols are handled.

Specific comments

#	Reviewer comment	Editorial comment
1	Despite its potential utility for sequencing and bioinformatics service facilities, UniverSC does not show any novelty, and is a simple wrapper around existing tools. Moreover, the authors don't mention that STARsolo can also perform these tasks, without the need for the heavy CellRanger pipeline. The manuscript seems therefore of very limited interest.	**Please clearly delineate how UniverSC is different/ superior to STARsolo.**

Reviewer #3
Report received: 21 March 2021

**Reviewer #3**	This reviewer has not chosen to waive anonymity. The reviewer's identity can only be shared with representatives of an established journal editorial office.
**Reviewer #3 expertise** *Summarised by the editor*	This reviewer is an expert on computational tools for single cell RNA-seq analysis
**Editor's comments about this review**	This reviewer acknowledges the utility of UniverSC platform in the comparison and evaluation across data generated from different platforms, but feels that testing it on more datasets can make the conclusions more robust, to which we agree. There are other important clarifications and methodological details which we would like to be addressed.

Reviewer #3 comments	
**Overview**	Kelly et al. developed a universal single-cell data processing tool named UniverSC, which could support any UMI-based platform. UniverSC may be useful for the integration, comparison, and evaluation across data generated from different platforms.

Specific comments

#	Reviewer comment	Editorial comment
1	**Comments on Impact:**<br><br>UniverSC could benefit the single-cell sequencing data analysis.	
2	UniverSC could be a useful tool for single-cell sequencing data processing. I have the following concerns:<br><br>1. The authors only tested the performance of UniverSC on three datasets. If more datasets could be tested, their results/conclusions will be more solid.<br><br>2. Does UniverSC have limit in the number of cells for processing? If yes, what is the largest number of cells can be analyzed?<br><br>3. What is the speed for UniverSC handling the single-cell sequencing data?<br><br>4. How much memory does UniverSC need to process the single-cell sequencing data with 10,000 cells?	**Please test for more datasets as mentioned and address other comments.**
3	**Comments on Reproducibility:**<br><br>Could the authors provide more details about how they process the test datasets in the Methods section? For example, how many cells does each dataset have? Did they filter any cells or use all the cells of each dataset in the analysis?	**Please provide more methodological details as mentioned.**

Open Research Evaluation	
**Data Availability**	
**Data Availability Statement**	Publication in any Nature Portfolio journal (and many other journals) requires a Data Availability statement that provides details on whether and how all data associated with the study can be accessed. If any restrictions apply for accessing any associated datasets, this should be specified in the Data Availability statement. See our policy page for details.
**Source data**	Source data files will be mandatory prior to publication in a Nature Portfolio journal.<br>All sets of processed data (gene-barcode matrices) are provided as supplemental data alongside with the manuscript. There are also references of supplementary data or supplementary files 1 to 6 in the manuscript; however, supplementary data or files 1 to 6 have not been provided and hence could not be evaluated. Kindly provide them with the resubmission.
**Code Availability**	
**Code Availability**	Please note that the DockerHub web-link provided in the 'data' section of the reporting summary (https://hub.docker.com/repository/docker/tomkellygenetics/universc) is currently not accessible as login credentials are required to access the data. To ensure a rigorous review process, we ask that you provide the reviewers with login credentials.<br>At the time of publication, all custom software/code must be made publicly available. Please provide the GitHub and DockerHub web-links in the manuscript under the 'code availability' section as well as in the 'Software and code' section of the reporting summary. See our policy page for details.
**Code Citation**	In addition to making the custom code available, we ask that

	you ensure that the version of the code/software described in the paper is deposited in a DOI-minting repository (eg, Zenodo) and that this DOI is also cited in the main Reference list. See here for details.
**Research ethics**	
**Competing Interests**	In the interests of transparency and to help readers form their own judgements of potential bias, Nature Portfolio journals require authors to declare any competing financial and/or non-financial interests in relation to the work described.<br><br>Please provide a 'Competing interests' statement using one of the following standard sentences:<br><br>• The authors declare the following competing interests: [specify competing interests]<br><br>• The authors declare no competing interests.<br><br>See our competing interests policy for further information: https://www.nature.com/nature-research/editorial-policies/competing-interests
**Other notes**	
**Manuscript format**	The manuscript is currently in Brief Communications format. Please note that neither *Nature Communications* nor *Communications Biology* supports this format. We encourage you to format the paper with a full Introduction, Results, Discussion, and Methods section. Please note that we allow unlimited space for the Methods.
