# Supplementary material for: A flexible cross-platform single-cell data processing pipeline
Source: Nat Commun. 2022 Nov 11;13:6847. doi: 10.1038/s41467-022-34681-z (PMC9652453; doi:10.1038/s41467-022-34681-z)
Supplement: Supplementary file 6 — File S1, File S2, File S3, File S4, File S5, File S6, File S7, File S8, File S9, File S10, File S11 [file 41467_2022_34681_MOESM6_ESM.zip › UniverSC-Supplementary Files/FileS4_dropseqpipe0.6.zip!/dropseqpipe0.6/plots/violinplots_comparison_UMI.pdf]

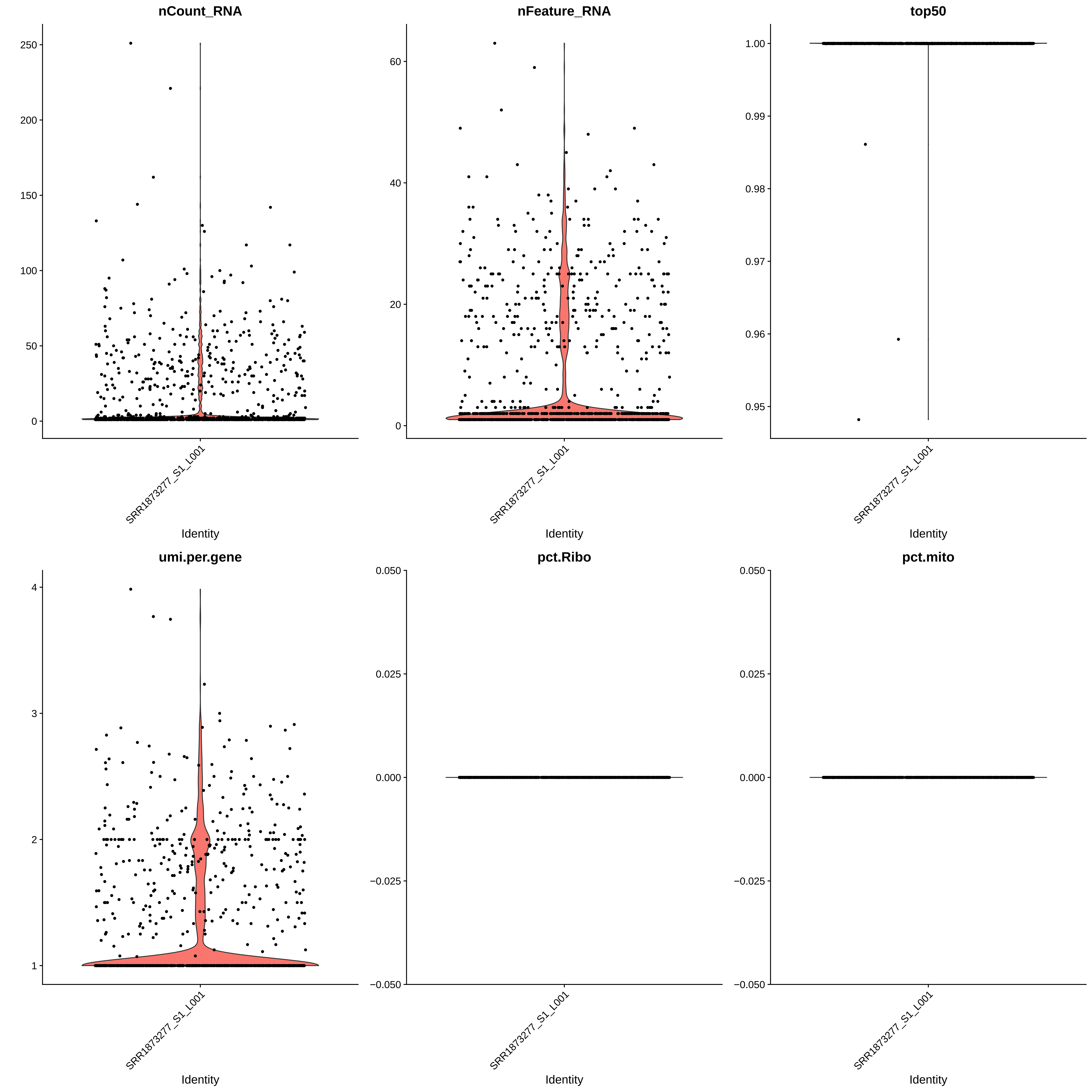
nCount_RNA
nFeature_RNA
top50
umi.per.gene
pct.Ribo
pct.mito
SRR1873277_S1_L001
Identity
0
50
100
150
200
250
20
40
60
0.95
0.96
0.97
0.98
0.99
1.00
1
2
3
4
−0.050
−0.025
0.000
0.025
0.050
