# Supplementary material for: A flexible cross-platform single-cell data processing pipeline
Source: Nat Commun. 2022 Nov 11;13:6847. doi: 10.1038/s41467-022-34681-z (PMC9652453; doi:10.1038/s41467-022-34681-z)
Supplement: Supplementary file 6 — File S1, File S2, File S3, File S4, File S5, File S6, File S7, File S8, File S9, File S10, File S11 [file 41467_2022_34681_MOESM6_ESM.zip › UniverSC-Supplementary Files/FileS4_dropseqpipe0.6.zip!/dropseqpipe0.6/plots/Count_vs_gene.pdf]

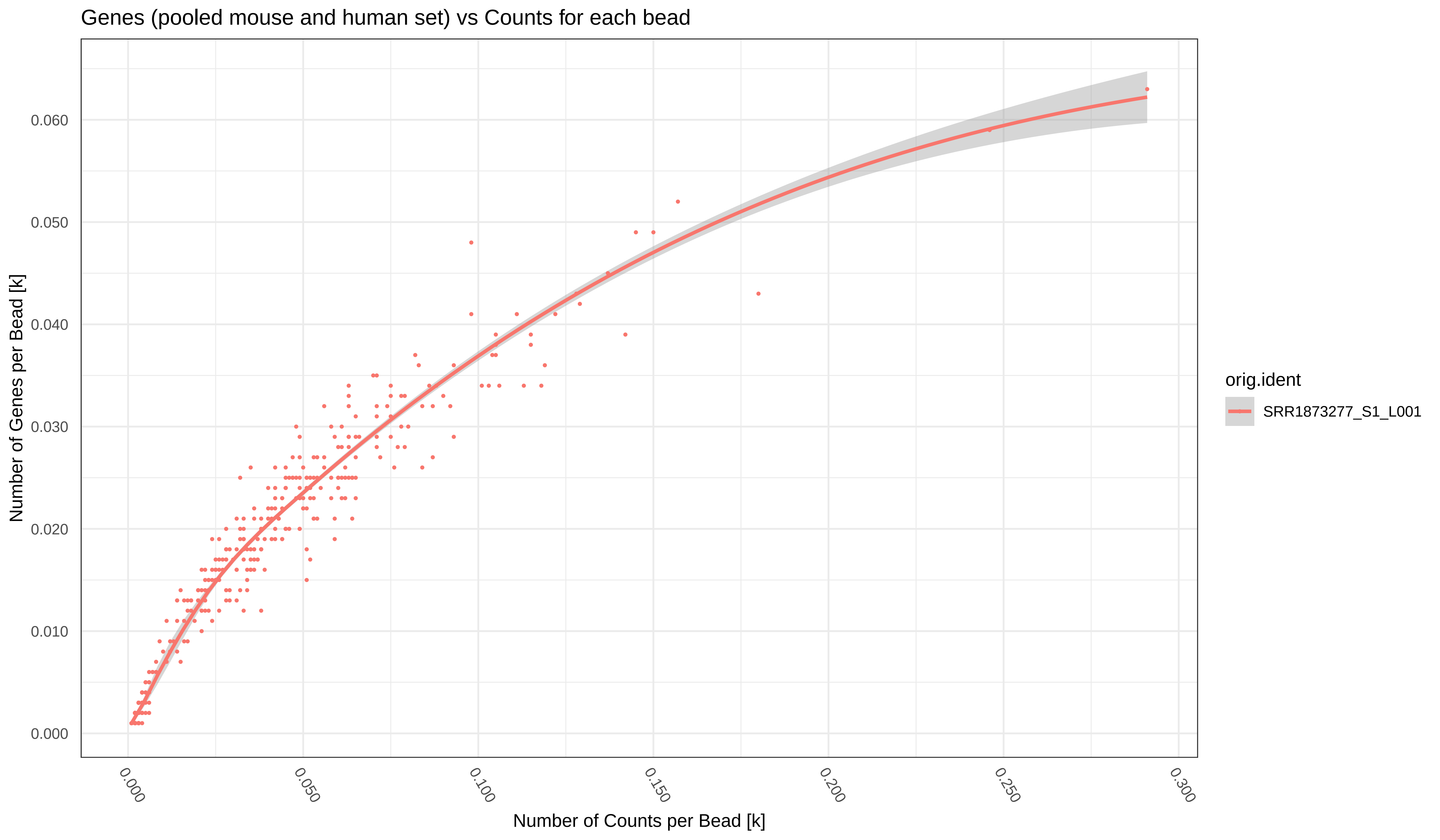
Genes (pooled mouse and human set) vs Counts for each bead
Number of Genes per Bead [k]
0.000
0.010
0.020
0.030
0.040
0.050
0.060
Number of Counts per Bead [k]
0.000
0.050
0.100
0.150
0.200
0.250
0.300
orig.ident
SRR1873277_S1_L001
