# Supplementary material for: A flexible cross-platform single-cell data processing pipeline
Source: Nat Commun. 2022 Nov 11;13:6847. doi: 10.1038/s41467-022-34681-z (PMC9652453; doi:10.1038/s41467-022-34681-z)
Supplement: Supplementary file 6 — File S1, File S2, File S3, File S4, File S5, File S6, File S7, File S8, File S9, File S10, File S11 [file 41467_2022_34681_MOESM6_ESM.zip › UniverSC-Supplementary Files/FileS4_dropseqpipe0.6.zip!/dropseqpipe0.6/logs/fastqc/SRR1873277_S1_L001_R1_fastqc.html]

SRR1873277\_S1\_L001\_R1\_001.fastq.gz FastQC Report 

FastQC Report

Fri 12 Jun 2020  
SRR1873277\_S1\_L001\_R1\_001.fastq.gz

## Summary

- Basic Statistics
- Per base sequence quality
- Per tile sequence quality
- Per sequence quality scores
- Per base sequence content
- Per sequence GC content
- Per base N content
- Sequence Length Distribution
- Sequence Duplication Levels
- Overrepresented sequences
- Adapter Content

## Basic Statistics

| Measure | Value |
| --- | --- |
| Filename | SRR1873277\_S1\_L001\_R1\_001.fastq.gz |
| File type | Conventional base calls |
| Encoding | Sanger / Illumina 1.9 |
| Total Sequences | 29265 |
| Sequences flagged as poor quality | 0 |
| Sequence length | 20 |
| %GC | 51 |

## Per base sequence quality

## Per tile sequence quality

## Per sequence quality scores

## Per base sequence content

## Per sequence GC content

## Per base N content

## Sequence Length Distribution

## Sequence Duplication Levels

## Overrepresented sequences

No overrepresented sequences

## Adapter Content

Produced by FastQC (version 0.11.8)
