# Supplementary material for: A flexible cross-platform single-cell data processing pipeline
Source: Nat Commun. 2022 Nov 11;13:6847. doi: 10.1038/s41467-022-34681-z (PMC9652453; doi:10.1038/s41467-022-34681-z)
Supplement: Supplementary file 6 — File S1, File S2, File S3, File S4, File S5, File S6, File S7, File S8, File S9, File S10, File S11 [file 41467_2022_34681_MOESM6_ESM.zip › UniverSC-Supplementary Files/FileS4_dropseqpipe0.6.zip!/dropseqpipe0.6/plots/knee_plots/SRR1873277_S1_L001_knee_plot.pdf]

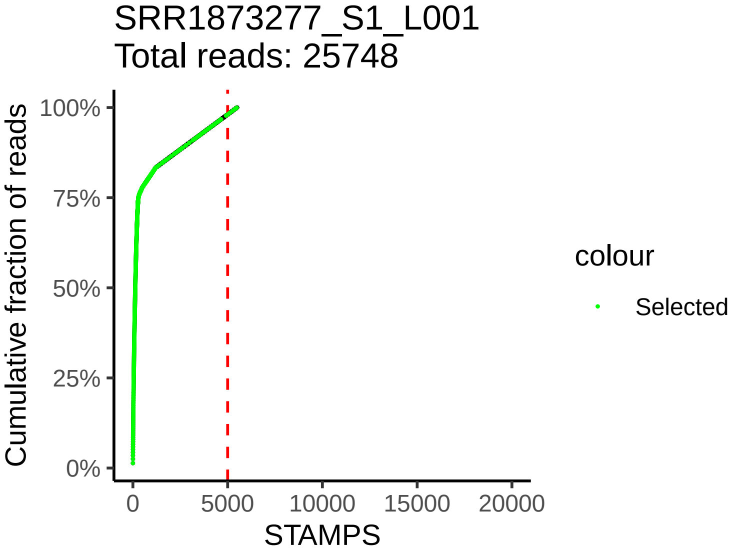

SRR1873277_S1_L001
Total reads: 25748
Cumulative fraction of reads
100%
75%
50%
25%
0%
0
5000
10000
15000
20000
STAMPS
colour
Selected
